# Extensive Characterization of Mesenchymal Stem Cell Marker Expression on Freshly Isolated and *In Vitro* Expanded Human Adipose-Derived Stem Cells from Breast Cancer Patients

**DOI:** 10.1155/2020/8237197

**Published:** 2020-06-18

**Authors:** Premrutai Thitilertdecha, Visnu Lohsiriwat, Poonsin Poungpairoj, Varangkana Tantithavorn, Nattawat Onlamoon

**Affiliations:** ^1^Siriraj Research Group in Immunobiology and Therapeutic Sciences, Faculty of Medicine Siriraj Hospital, Mahidol University, 2 Wanglang Road, Bangkoknoi, Bangkok, Thailand; ^2^Biomedical Research Incubator Unit, Research Group and Research Network Division, Research Department, Faculty of Medicine Siriraj Hospital, Mahidol University, 2 Wanglang Road, Bangkoknoi, Bangkok 10700, Thailand; ^3^Department of Surgery, Faculty of Medicine Siriraj Hospital, Mahidol University, 2 Wanglang Road, Bangkoknoi, Bangkok, Thailand

## Abstract

Variation in numbers and functions of cells in fat tissues may affect therapeutic outcomes and adverse events after autologous fat tissue grafting in postmastectomy breast cancer patients; however, the relevant information regarding cellular components is still incomplete. Phenotypic characterization of heterogeneous cell subsets in stromal vascular fraction (SVF) isolated from fat tissues by flow cytometry was also limited to a combination of few molecules. This study, therefore, developed a polychromatic staining panel for an in-depth characterization of freshly isolated SVF and expanded adipose-derived stem cells (ADSC) from the patients. ADSC were found predominant in SVF (~65% of CD45^−^ cells) with a homogenous phenotype of CD13^+^CD31^−^CD34^+^CD45^−^CD73^+^CD90^+^CD105^−^CD146^−^ (~94% of total ADSC). Endothelial progenitor cells (EPC) and pericytes were minor (~18% and ~11% of CD45^−^ cells, respectively) with large heterogeneity. Downregulation of CD34 and upregulation of CD105 in ADSC were profound at passage 3, showing a phenotype similar to the classical mesenchymal stem cells from the bone marrow. Results from this study demonstrated that fat tissue collected from patients contains ADSC with a highly homogenous phenotype. The *in vitro* culture of these cells maintained their homogeneity with modified CD34 and CD105 expression, suggesting the expansion from a single population of ADSC.

## 1. Introduction

White adipose tissue has been recognized as the alternative source for stromal precursors and stem cells. Normally, adipose tissues can be divided into two types including white and brown adipose tissues according to their morphology and physiology. White adipose tissue contains a single lipid droplet creating white to yellow appearance and functions by storing lipids for excessive energy, whereas brown adipose tissue comprises multiple small vacuoles with abundance of iron-containing mitochondria generating brown color and works through lipid burning for heat production [1–3]. Besides these dissimilarities, brown adipose tissue is less in quantity in adult humans and located in vital regions such as cervical, supraclavicular, and axillary [4]. White adipose tissue is found predominantly in subcutaneous and several visceral depots (e.g., abdomen, hip, and thigh); thus, it becomes a sensible source for progenitor stem cells.

Compared to the bone marrow—another recommended source of stem cells, the yield of mesenchymal stem cells (MSC) from white adipose tissue was able to reach 0.5–1.25 × 10^6^ cells/gram adipose tissue [5, 6] while only 0.001–0.01% of isolated cells was averagely achieved from the bone marrow [7] which was remarkably lower and insufficient for further propagation to use in cell therapy. The harvesting procedure of these bone marrow-derived stem cells (BMSC) is also relatively invasive to the patients and costs higher. Although BMSC are considered as a gold standard for adult stem cells, several concerns previously mentioned have become its limitation for clinical implementation. Other types of stem cells including embryonic stem cells (ESC) and induced-pluripotent stem cells (iPSC) have been restricted for clinical practices due to ethical consideration and cell regulation. Therefore, adipose-derived stem cells (ADSC) have recently been more attractive for therapeutic potentials because of their less invasive harvesting technique, less expensive cost, greater yield, and confirmed multilineage differentiation ability the same as MSC characteristics [5, 6, 8, 9].

A heterogeneous population of stromal vascular fraction (SVF) containing vascular endothelial cells, endothelial progenitor cells (EPC), pericytes, infiltrating cells of hematopoietic lineage, and adipose-derived stem cells (ADSC) can be isolated from lipoaspirates by enzymatic digestion and mechanical processing [8, 10–13]. As ADSC are widely known for their regenerative property, they have then been introduced not only to reconstructive surgery targeting in soft tissues and skin but also in all fields of surgery with a wide range of potential clinical uses [14]. Oncoplastic breast surgery is one of the several surgical applications using ADSC through fat grafting for postmastectomy breast reconstruction in breast cancer patients [15–17]. The clinical outcomes rely on abilities of ADSC in proliferation and differentiation to new functional adipocytes together with maintenance of mature fat graft volume. Therefore, ADSC have become great potential for novel breast reconstruction approaches and attractive to recent tissue engineering [18] instead of BMSC which were reported to occupy higher differentiation tendency towards osteoblasts and chondrocytes than adipocytes [19]. Many issues regarding cellular biology, oncological safety, clinical efficacy, and cell production as well as surgery techniques and experience with procedure are then concerned.

A supportive use of ADSC for clinical applications such as cell-assisted lipotransfer (CAL) was introduced by using a combination of SVF and aspirated fat for autologous tissue transfer [20]. This CAL technique was able to increase the efficacy by showing the higher survival rate and persistence of transplanted fat when compared to non-CAL (i.e., aspirated fat alone without ADSC) as well as reduced adverse effects from calcification, fibrosis formation, and pseudocyst [20]. Aspirated fat was then served as injection material for soft tissue augmentation which was also rich in EPC and pericytes promoting angiogenesis and microvasculature. However, EPC were concerned for catalyzing tumor vascularization [21, 22]. Detailed identification of EPC and pericytes in lipoaspirates is then warranted for better understanding of their relationship with the partial necrosis of aspirate fat or cancer-promoting risk after fat transplant.

Therefore, phenotypic characterization of ADSC is essential as the initial step for cellular biology confirmation. Flow cytometry is widely used since it is the gold standard method for evaluation of cell composition in a sample and functionally relevant cell surface marker expressions. Although numerous studies put efforts by using a broad range of surface markers for ADSC identification, there is still a controversial discussion on the expression of some surface protein molecule by ADSC on the day of isolation, such as CD105 [23, 24] and CD146 [24–26]. This variation may be resulted from different monoclonal antibody panels used for multicolor staining in a single sample and from physiological difference between fresh and cryopreserved samples. Isolated SVF or ADSC samples were also obtained from healthy donors in most reports while the use of ADSC for postmastectomy breast reconstruction requires autologous fat transplantation. This study is thus aimed at investigating an in-depth characterization of ADSC, EPC, and pericytes in fresh liposuction aspirates from breast cancer patients by using a developed 8-color staining panel with flow cytometric analysis. Serial changes in ADSC phenotypic profiles were also explored from the day of cell isolation until completion at passage 3. Besides that, multilineage differentiation ability of the expanded ADSC was evaluated to ensure their MSC characteristics.

## 2. Materials and Methods

### 2.1. Study Population

Twenty-two breast cancer female patients aged between 47 and 62 years old requiring autologous fat transplantation for breast reconstruction at the Faculty of Medicine Siriraj Hospital, Mahidol University, Bangkok, Thailand, were recruited for the study. The protocols were approved by the Institution Review Board (IRB) of the Faculty of Medicine Siriraj Hospital (COA number 580/2016). Written informed consent was also obtained from each subject prior to the study.

### 2.2. Sample Collection

Lipoaspirates were withdrawn from the abdomen by a tumescence technique using a 3 mm diameter suction tube coupled with a 10 mL vacuum syringe. The collected tissues were then kept in the syringes for fresh isolation and characterization of ADSC on the same day before further serial passaging.

### 2.3. Cell Isolation from Lipoaspirates

Raw lipoaspirates were centrifuged at 2,000g for 3 min followed by a removal of oil and blood. The remaining fat tissues were digested with 2 mg/mL collagenase A type I (Gibco, Thermo Fisher Scientific, MA, USA) at 37°C for 60 min under shaking. Neutralization of enzymatic activity was done by adding a complete medium containing Dulbecco's modified Eagle's medium (DMEM, Gibco, Thermo Fisher Scientific, MA, USA), 10% fetal bovine serum (FBS, Gibco, Thermo Fisher Scientific, MA, USA), 1% l-glutamine (Gibco, Thermo Fisher Scientific, MA, USA), 1% penicillin-streptomycin (Pen-Strep, Gibco, Thermo Fisher Scientific, MA, USA), and 0.01% gentamycin (Gibco, Thermo Fisher Scientific, MA, USA) before centrifugation at 400g, 4°C for 10 min. After that, the collected pellet was resuspended in ammonium chloride potassium (ACK) lysis buffer (Gibco, Thermo Fisher Scientific, MA, USA) for red blood cell (RBC) lysis and centrifuged at 400g, 4°C for 10 min, for RBC removal. The obtained pellet was then resuspended in phosphate-buffered saline (PBS, Gibco, Thermo Fisher Scientific, MA, USA) containing 1% bovine serum albumin (BSA, Sigma-Aldrich, USA), and the cell suspension was filtered through 100 and 40 *μ*m cell strainers (Corning, NY, USA) to discard cellular debris followed by centrifugation again at 400g, 4°C for 10 min. The SVF pellet was collected and resuspended in 1% BSA in PBS. The cells were then counted by a trypan blue exclusion method.

### 2.4. Cell Culture

Three samples of freshly isolated SVF cells at the day of isolation (D0) were cultured in the DMEM complete medium at 10,000 cells/cm^2^ of a culture plate. After 3 days, nonadherent cells were washed and discarded. The remaining adherent cells were then expanded in complete medium, and replenishments of fresh medium were performed every 3 days. When the expanded cells reached 80% confluency, they were detached from the culture plates with 0.25% trypsin (Gibco Life Technologies, CA) and considered to be at passage 0 (P0). The isolated adherent cells at P0 were then continually expanded for subcultivation through passage 3 (P3). The expanded ADSC at each passage (i.e., P0, P1, P2, and P3) were counted by a trypan blue exclusion method and characterized by immunofluorescent staining and flow cytometric analysis.

### 2.5. Phenotypic Characterization

For phenotypic characterization of the freshly isolated and expanded cells, they were stained with fluorochrome-conjugated monoclonal antibodies including CD13-allophycocyanin (APC), CD31-Alexa Fluor® 488, CD34-Brilliant Violet™ 421 (BV421), CD45-peridinin-chlorophyll-protein (PerCP), CD73-phycoerythrin/Dazzle™ (PE/Dazzle™) 594, CD90-Brilliant Violet™ 510 (BV510), CD105-phycoerythrin cyanine 7 (PECy7), and CD146-phycoerythrin (PE). All reagents were obtained from BioLegend, CA, USA. The samples were then incubated for 15 min before washing and resuspending in 450 *μ*L PBS. All samples were analyzed by LSRFortessa flow cytometer (BD Biosciences, USA) and FlowJo® software (Tree Star, San Carlos, CA).

### 2.6. Multilineage Differentiation

At the end of passage 3, the cultured ADSC were confirmed for their differentiation capability towards adipocytes, osteocytes, and chondrocytes through histological analyses. For adipogenesis, the expanded ADSC at 40,000 cells/well in a 6-well plate were cultured in 1 mL of adipogenic differentiation medium (STEMCELL™ Technologies, Canada) and then incubated at 37°C and 5% CO_2_ for 14 days. On day 14, the induced cells were washed with 1X PBS twice and fixed with 10% formaldehyde in PBS for 30 min. After that, the fixed cells were washed with 60% isopropanol (Sigma-Aldrich, USA) in aqueous before stained with Oil Red O staining solution (Sigma-Aldrich, USA) for 60 min. The stained cells were extensively washed with water to remove unbound dye and subsequently observed under an inverted fluorescent microscope (Nikon, Ti-S Intensilight Ri1 NIS-D, Japan). Representative images of the induced cells were compared to a control group (i.e., noninduced ADSC cultured in DMEM complete medium).

With respect to osteogenesis, ADSC at 40,000 cells/well in a 6-well plate were cultured in 1 mL of osteogenic differentiation medium (STEMCELL™ Technologies, Canada) and then incubated at 37°C and 5% CO_2_ for 14 days. On day 14, the induced cells were washed with 1x PBS twice and fixed with 70% ethanol (Sigma-Aldrich, USA) for 30 min. After that, ethanol was removed and the fixed cells were stained with Alizarin Red S staining solution (Sigma-Aldrich, USA) for 30 min before extensively washed with water. Representative images of the induced cells were captured by the inverted fluorescent microscope and compared with a noninduction control.

For chondrogenesis, a micromass culture system was used. The cultured ADSC at concentration of 200,000 cells/10 *μ*L DMEM complete medium were dropped at the center of a 12-well plate and incubated at 37°C and 5% CO_2_ without the culture medium for 1 h. After that, the chondrogenic differentiation medium containing DMEM supplemented with 100 nM dexamethasone (Sigma-Aldrich, USA), 50 mg/mL ascorbic acid (Sigma-Aldrich, USA), 100 *μ*g/mL sodium pyruvate (Sigma-Aldrich, USA), 1 : 100 diluted ITS+Premix (a mixture containing 6.25 mg/mL insulin, 6.25 mg/mL transferrin, 6.25 mg/mL selenous acid, 1.25 mg/mL BSA, and 5.35 mg/mL linoleic acid, BD Biosciences, USA), 10 ng/mL transforming growth factor-beta 1 (TGF-*β*1, PeproTech®, USA), and 40 mg/mL proline (Sigma-Aldrich, USA) was added into the culture plate and incubated at 37°C and 5% CO_2_ for 21 days. The chondrogenic differentiation medium was changed every 3 days. On day 21, the cell pellets were frozen in Tissue-Tek® O.C.T.™ Compound (Sakura®, Japan) with liquid N_2_ for cryostat sectioning by CryoStar™ NX70 Cryostat (Thermo Fisher Scientific, USA). The sectioned cell samples were placed on microscope slides, then washed with 1x PBS thrice and fixed with 10% formaldehyde in PBS for 10 min before gently removing the fixing agent. The fixed cells were stained with Alcian Blue staining solution (Sigma-Aldrich, USA) for 30 min and, respectively, washed with water, 70%, 80%, and 90% and absolute ethanol and xylene. Representative images of the induced cells were captured by the inverted fluorescent microscope and compared with a -induction control.

### 2.7. Data Analysis

Statistical analyses were performed using GraphPad Prism® software version 7.02 (GraphPad Software, Inc., La Jolla, CA). Data was expressed as mean ± standard deviation (SD). A two-way analysis of variance with a Bonferroni's multiple comparisons test was used to determine statistical differences of the mean quantity among SVF subpopulations including mesenchymal stem cells (MSC), ADSC, EPC, and pericytes. *P* values < 0.05 were considered as a statistical significance.

## 3. Results

### 3.1. Characterization of Cell Populations in SVF of Lipoaspirates from Breast Cancer Patients

**Figure 1:**
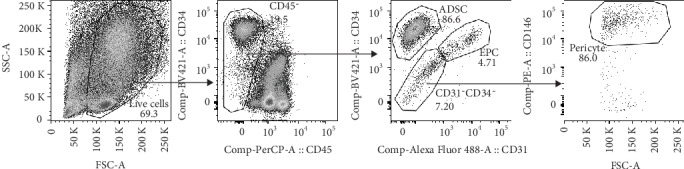
Representative gating strategy to identify ADSC, EPC, and pericytes in freshly isolated SVF.

To identify cell populations in SVF of white adipose tissues from breast cancer patients, an 8-marker staining panel including CD13, CD31, CD34, CD45, CD73, CD90, CD105, and CD146 was developed for polychromatic flow cytometric analysis. A gating strategy was employed for phenotypic characterization of cell subpopulation (Figure 1). Freshly isolated SVF cells were first gated for doublet discrimination (data not shown), and then, a live cell population was identified by using the light scattered properties (FSC-A vs. SSC-A). After that, nonhematopoietic cells (i.e., CD45^−^) were selected before being further identified into three subpopulations of SVF based on the expression of CD34 and CD31. ADSC were thus identified as CD31^−^CD34^+^, whereas EPC were identified as CD31^+^CD34^+^. In addition, CD31^−^CD34^−^ was further characterized by using the expression of CD146 to obtain pericytes (CD31^−^CD34^−^CD146^+^). According to this gating strategy, the minimum markers of CD31, CD34, CD45, and CD146 were sufficient to identify 3 major heterogeneous subsets of SVF.

**Figure 2:**
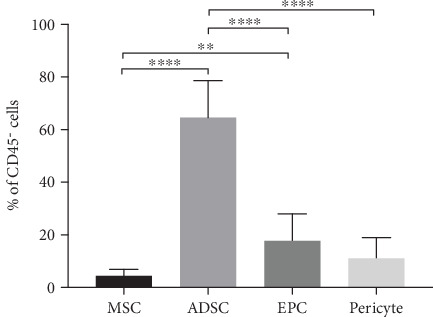
Percentages of freshly isolated SVF subsets including MSC, ADSC, EPC, and pericytes. All data are represented as mean ± SD (*n* = 22; ∗∗*P* < 0.01, ∗∗∗∗*P* < 0.0001).

Freshly isolated SVF samples from twenty-two patients were then used to determine the amount of each SVF subpopulations. In a live cell population, 22.9 ± 10.2% was identified as nonhematopoietic cells. This cell subset was further used to identify ADSC, EPC, and pericytes. Quantities of cell subpopulations in the CD31^−^CD34^−^ subset were also investigated for the expression of a set of surface markers including CD73, CD90, and CD105 (i.e., considered as MSC). As shown in Figure 2, the nonhematopoietic cell portion of SVF comprised a significantly large population of ADSC (64.6 ± 14.2%) followed by similar numbers of EPC and pericytes (17.8 ± 10.4% and 11.2 ± 7.8%, respectively). In contrast, only a small population of these cells was identified as MSC (4.5 ± 2.4%), suggesting a majority of stem cells in SVF belongs to ADSC.

### 3.2. Phenotypic Profiles and Subpopulations of ADSC, EPC, and Pericytes

**Figure 3:**
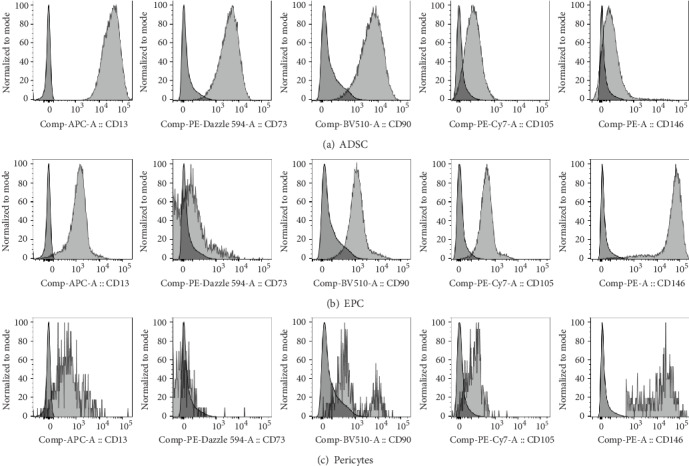
Representative overlaid histograms for phenotypic characterization of (a) ADSC, (b) EPC, and (c) pericytes when compared to unstained cells (darker color, on the left).

With respect to detailed phenotypic profiles of ADSC, EPC, and pericytes in the freshly isolated SVF, their surface maker expressions of CD13, CD73, CD90, CD105, and CD146 were determined (Figure 3). For ADSC, the expressions of CD13, CD73, and CD90 were observed with high intensity while the expression of CD105 and CD146 was not found. The surface marker expression of EPC, on the other hand, exhibited moderate expression intensities of CD13, CD90, and CD105 with a high intensity of CD146 and an absence of CD73 expression. For pericytes, the dim expressions of CD13 and CD90 were observed while the expression of CD146 was found with high intensity. The expressions of CD73 and CD105 were also not observed in pericytes. These results suggested that ADSC population was homogeneous, whereas EPC and pericytes were heterogeneous.

Subsets of ADSC, EPC, and pericytes were then examined based on these 5 surface markers by using Boolean gating analysis (Table 1). The result showed that a majority of ADSC exhibited CD13^+^CD73^+^CD90^+^CD105^−^CD146^−^ phenotype (93.6 ± 2%) and the rest of the ADSC population either expressed CD146 or CD105. Unlike ADSC, a great variation was observed for EPC subpopulations. Ten subpopulations of EPC were identified in which the majority exhibited CD13^+^CD73^−^CD90^+^CD105^+^CD146^+^ phenotype (33.7 ± 21.0%). Interestingly, approximately 15% of EPC showed simultaneous expressions of CD73, CD90, CD105, and 146 (11.7 ± 7.4% for the CD13^+^CD73^+^CD90^+^CD105^+^CD146^+^ subset and 3.1 ± 2.9% for the CD13^−^CD73^+^CD90^+^CD105^+^CD146^+^ subset). It is worth noting that the expressions of CD90 and CD146 were common in most EPC subsets with 8 out of 10 subsets expressing CD90 and 7 out of 10 subsets expressing CD146. For pericytes, since all of them expressed CD146, 8 subpopulations were characterized based on differential expressions of CD13, CD73, CD90, and CD105. The predominant subset exhibited CD13^+^CD73^−^CD90^+^CD105^−^CD146^+^ phenotype (26.7 ± 20.3%) and followed by the CD13^−^CD73^−^CD90^+^CD105^−^CD146^+^ subset (18.3 ± 20.6%). Interestingly, these 2 subsets contributed to almost half of pericytes with mere difference on CD13 expression.

### 3.3. Phenotypic Changes of ADSC after Serial Passaging

**Figure 4:**
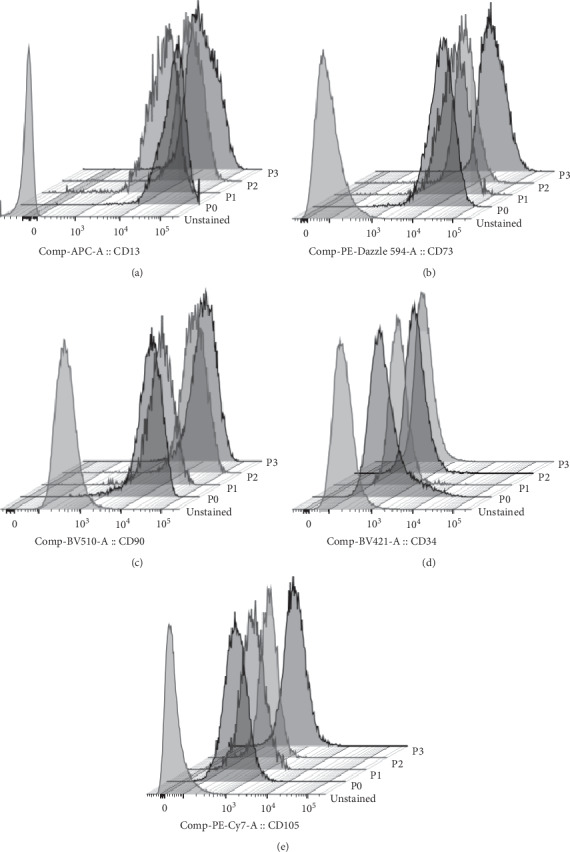
Representative stacked histograms of expanded ADSC at passages 0–3 for phenotypic changes when compared to unstained cells.

While ADSC on the day of isolation (D0) did not express CD105 which was regarded as a maker for identification of MSC (i.e., used in a combination with CD73 and CD90), the expression of CD34 was observed appearing to be similar to pericyte progenitors and hematopoietic stem cells. These results suggested that freshly isolated ADSC from SVF are distinct from MSC. Therefore, the phenotypic alteration during the *in vitro* expansion of ADSC was then determined. ADSC were expanded over serial passaging starting from the day of SVF isolation determined as passage 0 (P0) until the completion at passage 3 (P3). Phenotypes of the expanded ADSC from each passage were analyzed by polychromatic flow cytometry using the 8-marker staining panel. A representative phenotypic profile of the expanded ADSC from 4 different passages (P0, P1, P2, and P3) was demonstrated in Figure 4. Similar to freshly isolated ADSC from SVF, the expressions of CD13, CD73, and CD90 of the expanded ADSC remained consistently high throughout the culture period (Figure 4) while the expressions of CD45 and CD31 were absent from the expanded cell populations (data not shown). More importantly, a remarkable downregulation of CD34 was observed since P0 and its expression gradually decreased until a complete diminution on P3. On the contrary, most of the expanded cells showed a remarkable upregulation of CD105 since P0 and its expression maintained throughout the culture period.

To characterize cell subpopulations in the expanded cells, simultaneous expressions of cell surface markers were analyzed. Although the highly homogeneous ADSC with CD13^+^CD31^−^CD34^+^CD45^−^CD73^+^CD90^+^CD105^−^CD146^−^ phenotype were observed in freshly isolated SVF and largely contributed as major progenitor cells in the expansion culture, a notable decrease of this cell population was observed on P0 and its presence remained low throughout the culture period (Table 2). While many different cell subsets were observed in the expanded cell population due to a variation in cell surface marker expressions, approximately 80% belonged to only 2 major phenotypes including CD13^+^CD31^−^CD34^+^CD45^−^CD73^+^CD90^+^CD105^+^CD146^−^ and CD13^+^CD31^−^CD34^−^CD45^−^CD73^+^CD90^+^CD105^+^CD146^−^ (Table 2). Interestingly, the frequency of the expanded cells with CD13^+^CD31^−^CD34^−^CD45^−^CD73^+^CD90^+^CD105^+^CD146^−^ phenotype was slightly higher than another subset at the beginning on P0 and then considerably increased over the serial passages (approximately 70% on P3). Due to these phenotypic changes, the characteristic of the expanded ADSC (i.e., CD13^+^CD34^−^CD45^−^CD73^+^CD90^+^CD105^+^CD146^−^) at the end of P3 became more similar to that of MSC from the bone marrow.

### 3.4. Differentiation Potential of the Expanded ADSC

**Figure 5:**
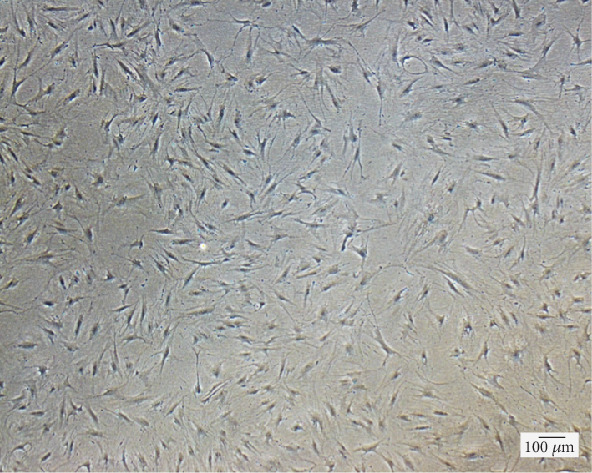
Representative image of the expanded ADSC with fibroblast-like appearance. The image was observed using the inverted fluorescent microscope (4x magnification, scale bar of 100 *μ*m).

**Figure 6:**
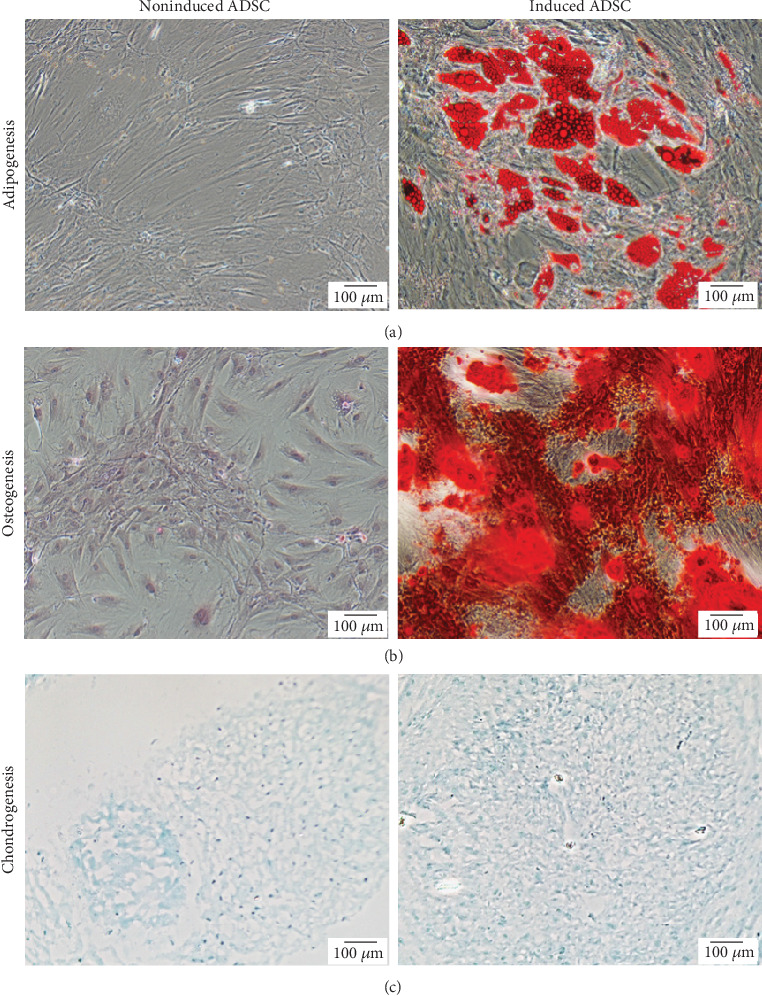
Representative images of multilineage differentiation ability of the expanded ADSC at passage 3. Cell histology in 2-dimentional culture of the induced cells was compared to that of the noninduced cells after different induction conditions: (a) adipogenic differentiation for 14 days and staining with Oil Red O; (b) osteogenic differentiation for 14 days and staining with Alizarin Red S; and (c) chondrogenic differentiation for 21 days and staining with Alcian Blue. All samples were observed using the inverted fluorescent microscope (10x magnification, scale bars: 100 *μ*m).

Before the evaluation of differentiation capability of the expanded ADSC at the end of P3, morphology of ADSC was observed for confirmation of a typical fibroblast-like adherent appearance (Figure 5). ADSC were then examined for their multipotency through histological analyses using specific induction and staining protocols. The induced and noninduced cells were stained with Oil Red O to detect neutral triglycerides and lipid droplets in adipogenic assay, whereas Alizarin Red S was used to identify calcium deposits in osteogenic detection and Alcian Blue was used to observe proteoglycan in chondrogenic protocol. Results show that ADSC were able to differentiate into adipocytes and osteocytes within 14 days as well as chondrocytes within 21 days under the induction conditions when compared to the noninduced ADSC (Figure 6).

## 4. Discussion

Our study demonstrates the existence of cell subpopulations within multipotent ADSC, EPC, and pericytes in freshly isolated SVF from abdominal fat of breast cancer patients. At present, characterization of the phenotypes and properties of ASDC, EPC, and pericytes is usually limited to SVF from healthy donors and only a few studies reported cell subpopulations in freshly isolated SVF from breast cancer patients [21, 22, 27]. Most previous cellular characterization was performed through multiparameter immunophenotypic analysis with a single marker or a combination of up to 4 biomarkers [24, 25, 27–29] which may be inadequate to clearly clarify subpopulations. We then developed the 8-color staining panel with flow cytometry for extensive and accurate characterization of ADSC, EPC, and pericytes with their cellular subsets in lipoaspirates from breast cancer patients. The typical MSC marker proteins including surface enzymes CD13 (amino-peptidase) and CD73 (5′ecto-nucleotidase) and extracellular matrix proteins CD90 (Thy-1), CD105 (endoglin), and CD146 (Muc18) as well as hematopoietic cell lineage markers CD34 (mucusialin) and CD45 (leukocyte common antigen, LCA, Ly-5), and endothelial cell marker CD31 (platelet endothelial cell adhesion molecule-1, PECAM-1) were chosen for immunophenotypic identification.

Our results showed that ADSC, EPC, and pericytes were able to be distinguished by a minimum of 4 markers including CD31, CD34, CD45, and CD146. While all three cell types do not express CD45, ADSC only expressed CD34 and EPC expressed both CD31 and CD34. Pericytes, on the other hand, did not express all of those markers except CD146. This identification of the 3 main populations was in agreement with previous studies [22, 26, 27, 30]. In our freshly isolated SVF, ADSC were the most abundant followed by EPC and pericytes (64.6 ± 14.2%, 17.8 ± 10.4%, and 11.2 ± 7.8% of CD45^−^ cells, respectively) which differed from a finding by Agostini *et al*. showing ADSC with 58.8 ± 16.6% and EPC with 43.2 ± 16.6% of CD34^+^CD45^−^ cells [27]. The differences in numbers may be influenced from different presentation in cell percentages (% of CD45^−^ cells vs. % of CD34^+^CD45^−^ cells), variation in sample sizes (*n* = 22 vs. *n* = 6), and donor-dependent variability, despite the same sample sources from breast cancer patients and similar surface markers used for characterization. Moreover, most of our nonhematopoietic lineage (CD45^−^) in SVF comprised CD34^+^ cells (over 80% of CD45^−^ cells), meaning that percentages of ADSC and EPC would be even greater when reporting in % of CD34^+^CD45^−^ cells. By presenting cell numbers in % of CD45^−^ cells, it allows us to identify pericytes residing in CD34^−^CD45^−^ population.

Unlike MSC expressing CD73, CD90, and CD105, the expression of CD105 was absent from freshly isolated ADSC. In order to determine the presence of predefined MSC, we then examined whether there was any cells with CD34^−^CD45^−^CD73^+^CD90^+^CD105^+^ phenotype in freshly isolated SVF. Since a small population of MSC was found at 4.5 ± 2.4% of CD45^−^ cells, the result suggested that a larger proportion of ADSC might play a major role for regenerative effects after lipotransfer. To verify homogeneity and heterogeneity of the 3 main populations of SVF (including ADSC, EPC, and pericytes), a set of surface markers including CD13, CD73, CD90, CD105, and CD146 was taken into consideration. Freshly isolated ADSC (CD31^−^CD34^+^CD45^−^) were homogeneous as a major population belongs to cells with CD13^+^CD73^+^CD90^+^CD105^−^CD146^−^ phenotype (93.6 ± 2% of ADSC). In addition, two minor subsets with changes in either CD146 or CD105 expression (2.5 ± 1.6% and 1.7 ± 1.0% of ADSC, respectively) were identified. The presence of a large proportion of the highly homogeneous ADSC suggested homogeneity in the regenerative capacity and served as a major source for the *in vitro* expanded ADSC.

On the contrary, EPC and pericytes were heterogeneous with the presence of several subsets. A majority of EPC was identified as CD13^+^CD73^−^CD90^+^CD105^+^CD146^+^ cells with the greatest number of 33.7 ± 21.0% of EPC and smaller proportions were distributed among the other 9 subsets expressing different combinations of those 5 markers. While pericytes were identified as the cells with CD31^−^CD34^−^CD45^−^CD146^+^ phenotype, almost a half portion of pericytes was identified as the cells with CD13^+^CD73^−^CD90^+^CD105^−^CD146^+^ (26.7 ± 20.3%) and CD13^+^CD73^−^CD90^+^CD105^−^CD146^+^ (18.3 ± 20.6%) phenotypes. The other 7 subsets of pericytes showed variation in the expressions of CD13, CD73, CD90, and CD105. Agostini *et al*. also reported a similar profiling of freshly isolated ADSC from breast cancer patients; however, EPC were found with the different phenotype of CD13^−^ and CD73^+^ [27]. These differences may be caused from a different combination of cell surface markers used in the study as a maximum of 4 surface markers was analyzed simultaneously (i.e., combinations of CD34, CD45, and 7-aminoactinomycin (7-AAD) with either one of these markers including CD31, CD73, CD90, CD105, or CD146). This limitation in multicolor flow cytometry prevented simultaneous detection of different markers as observed in our study. We found the CD13^−^CD73^+^CD90^+^CD105^+^CD146^+^ cell subset with only 3.1 ± 2.9% of total EPC which was not represented as the majority of EPC. Nevertheless, the considerable phenotypes of all ADSC, EPC, and pericytes from breast cancer patients in this study were comparable to those from healthy donors [23].

In this study, phenotypic changes of the expanded ADSC over serial passaging were also observed. Although freshly isolated ADSC began with CD34^+^CD45^−^CD73^+^CD90^+^CD105^−^CD146^−^ phenotype, this population disappeared since the beginning passage (P0), suggesting the phenotypic differentiation occurs along the expansion of ADSC since the early stage of the *in vitro* culture. The results also showed that the *in vitro* expanded ADSC still maintained CD13^+^CD31^−^CD45^−^CD73^+^CD90^+^CD146^−^ phenotype, although the CD34 expression was gradually downregulated together with a marked increase in CD105 expression since P0 which maintained at high levels over the culture period as previously described [6, 27]. Most of all expanded ADSC remarkably and stably exhibited CD34^−^ and CD105^+^ phenotypes at passage 3 (P3) which were in agreement with those reported by Agostini *et al*. [27].

More importantly, the characteristic of a large proportion of the expanded ADSC (CD13^+^CD31^−^CD34^−^CD45^−^CD73^+^CD90^+^CD105^+^CD146^−^) became similar to MSC characteristic from the bone marrow (CD34^−^CD45^−^CD73^+^CD90^+^CD105^+^) [31]. However, a small proportion of the expanded ADSC with CD13^+^CD31^−^CD34^+^CD45^−^CD73^+^CD90^+^CD105^+^CD146^−^ phenotype was detected. Although this subpopulation expressed CD34 similarly to freshly isolated ADSC, the upregulation of CD105 was observed, suggesting the presence of an intermediary differentiated subset that may later downregulate CD34 expression. Furthermore, the phenotype of the cultured ADSC from breast cancer patients was similar to that from healthy donors [28, 32], suggesting that regenerative functions of ADSC and MSC should be indifferent after the expansion. This can be partly supported by the evidence showing that the cultured ADSC from the breast cancer patients possessed the same multipotent differentiation ability towards adipocytes, osteocytes, and chondrocytes. Therefore, the observed phenotypic comparability can at least affirm the use of ADSC from breast cancer patients for reconstructive surgery. Despite the flow cytometric staining strategy for immunophenotypic characterization of ADSC used in this study, the omics methodology has also been an alternative approach for further molecular identification of MSC to distinguish MSC between different cell sources and cell subtypes by using ribonucleic acid (RNA) deep sequencing together with nano-liquid chromatography (LC)-mass spectrometry (MS)/MS analyses [33]. However, this latter approach is more complicated and more expensive as well as has several concerns on possible overestimation of differences from biological noise in RNA sequencing technique and dynamic range restriction in MS methods.

Regarding the clinical translation in postmastectomy breast reconstruction, there are two tentative approaches for lipoinjection by using either freshly isolated SVF or cultured ADSC together with aspirated fat for soft tissue augmentation and tissue defect restoration. Sufficient numbers of ADSC in freshly isolated SVF without *in vitro* expansion can be achieved for treatment if a large volume of liposuction aspirates can be collected from adipose tissue sources. Alternatively, a selection of specific cell subsets with clinical relevance for treatment may offer a beneficial option. The results from our study provide promising criteria for the characterization of specific cell subsets which might result in a superior outcome for tissue reconstruction. Although ADSC in freshly isolated SVF have been considered as minimal manipulated cells providing more safety than the cultured ones, their enrichment may be required to reach therapeutic numbers in some cases, such as patients with low fat tissue, underweight patients, or patients with extensive reconstruction procedures. Moreover, the usefulness of liposuction aspirates containing EPC and pericytes remains controversial whether providing advantages in therapeutic support or disadvantages in cancer promotion [34, 35]. It is worth noting that freshly isolated and long-term cryopreserved ADSC themselves unlikely caused tumorigenesis and the cryopreserved ADSC were able to maintain normal levels of tumor suppressor markers, telomerase activity, and telomere length without serious DNA damage throughout 3 months of cryopreservation [36]. Identification of cellular components in SVF is then necessary and should be employed to ensure phenotypes and functional characteristics of ADSC, EPC, and pericytes in lipoaspirates used in a reconstruction procedure. Besides using ADSC for breast reconstruction, ADSC have also been extensively studied for other therapeutic potentials such as ischemic disease therapy through the paracrine secretion of bioactive factors in order to promote tissue repair and angiogenesis [37], impairment of irradiated wounds via wound healing acceleration and tissue revitalization [38], treatment of peripheral nerve injuries by promoting axon regeneration, myelin formation, and restoration of denervation muscle atrophy [39], and treatment of avascular necrosis of femoral head (AVNFH) via the increase in vascularity and new bone formation [40]. These various clinical applications thus make ADSC a promising therapeutic candidate for advanced cell-based therapy in many diseases.

## 5. Conclusion

In this study, we determined the in-depth characteristics of ADSC, EPC, and pericytes together with their subsets in freshly isolated SVF from 22 breast cancer patients by using our developed multiparametric phenotyping based on flow cytometric analysis using 8 surface protein markers. Phenotypic changes and multipotent differentiation capability of the expanded ADSC were also confirmed and similar to MSC characteristic. Comprehensive phenotypes of SVF observed in this study may associate with clinical outcomes, survival rate of transferred fat tissue, and cancer-promoting risk after postmastectomy breast reconstruction. The identification of functional relevant cell surface markers of individual cellular subset is also important for further investigation to determine its contribution in therapeutic functions or adverse side effects.

## Figures and Tables

**Table 1:** Percentages (mean ± SD) of subpopulations in ADSC, EPC, and pericytes (*n* = 22).

Population	Subset	% Frequency of total population
ADSC	CD13^+^CD73^+^CD90^+^CD105^−^CD146^−^	93.6 ± 2.0
	CD13^+^CD73^+^CD90^+^CD105^−^CD146^+^	2.5 ± 1.6
	CD13^+^CD73^+^CD90^+^CD105^+^CD146^−^	1.7 ± 1.0

EPC	CD13^+^CD73^−^CD90^+^CD105^+^CD146^+^	33.7 ± 21.0
	CD13^+^CD73^+^CD90^+^CD105^+^CD146^+^	11.7 ± 7.4
	CD13^−^CD73^−^CD90^+^CD105^−^CD146^+^	11.1 ± 18.3
	CD13^−^CD73^−^CD90^+^CD105^+^CD146^+^	9.3 ± 14.2
	CD13^−^CD73^−^CD90^−^CD105^−^CD146^−^	8.9 ± 13.9
	CD13^+^CD73^−^CD90^+^CD105^−^CD146^+^	7.7 ± 7.1
	CD13^−^CD73^−^CD90^+^CD105^−^CD146^−^	6.4 ± 6.4
	CD13^−^CD73^+^CD90^+^CD105^+^CD146^+^	3.1 ± 2.9
	CD13^−^CD73^−^CD90^−^CD105^−^CD146^+^	1.2 ± 1.3
	CD13^−^CD73^−^CD90^+^CD105^+^CD146^−^	1.1 ± 3.2

Pericyte	CD13^+^CD73^−^CD90^+^CD105^−^CD146^+^	26.7 ± 20.3
	CD13^−^CD73^−^CD90^+^CD105^−^CD146^+^	18.3 ± 20.6
	CD13^−^CD73^−^CD90^−^CD105^+^CD146^+^	5.9 ± 6.8
	CD13^+^CD73^−^CD90^−^CD105^−^CD146^+^	5.3 ± 4.2
	CD13^+^CD73^−^CD90^+^CD105^+^CD146^+^	4.7 ± 5.5
	CD13^+^CD73^+^CD90^+^CD105^−^CD146^+^	1.9 ± 1.3
	CD13^−^CD73^−^CD90^+^CD105^+^CD146^+^	1.8 ± 2.3
	CD13^+^CD73^+^CD90^+^CD105^+^CD146^+^	1.5 ± 1.4

**Table 2:** Percentages (mean ± SD) of subpopulations in cultured ADSC over serial passaging from passages 0 to 3 (*n* = 3).

Cultured ADSC subset	P0	P1	P2	P3
CD13^+^CD31^−^CD34^+^CD45^−^CD73^+^CD90^+^CD105^−^CD146^−^	2.3 ± 0.5	1.8 ± 1.7	0.8 ± 1.0	0.7 ± 0.9
CD13^+^CD31^−^CD34^+^CD45^−^CD73^+^CD90^+^CD105^+^CD146^−^	37.5 ± 7.8	29.3 ± 12.6	17.9 ± 8.1	10.0 ± 6.2
CD13^+^CD31^−^CD34^−^CD45^−^CD73^+^CD90^+^CD105^+^CD146^−^	48.6 ± 6.7	50.9 ± 8.6	66.6 ± 2.7	71.8 ± 6.8

## Data Availability

All data generated or analyzed during this study are included in this published article.
